# *EjMYB8* Transcriptionally Regulates Flesh Lignification in Loquat Fruit

**DOI:** 10.1371/journal.pone.0154399

**Published:** 2016-04-25

**Authors:** Wen-qiu Wang, Jing Zhang, Hang Ge, Shao-jia Li, Xian Li, Xue-ren Yin, Donald Grierson, Kun-song Chen

**Affiliations:** 1 Zhejiang Provincial Key Laboratory of Horticultural Plant Integrative Biology, Zhejiang University, Zijingang Campus, Hangzhou, P.R. China; 2 The State Agriculture Ministry Laboratory of Horticultural Plant Growth, Development and Quality Improvement, Zhejiang University, Zijingang Campus, Hangzhou, P.R. China; 3 Plant & Crop Sciences Division, School of Biosciences, University of Nottingham, Sutton Bonington Campus, Loughborough, United Kingdom; Key Laboratory of Horticultural Plant Biology (MOE), CHINA

## Abstract

Transcriptional regulatory mechanisms underlying lignin metabolism have been widely studied in model plants and woody trees, but seldom in fruits such as loquat, which undergo lignification. Here, twelve *EjMYB* genes, designed as *EjMYB3-14*, were isolated based on RNA-seq. Gene expression indicated that *EjMYB8* and *EjMYB9* were significantly induced in fruit with higher lignin content resulting from storage at low temperature (0°C), while two treatments (low temperature conditioning, LTC; heat treatment, HT) both alleviated fruit lignification and inhibited *EjMYB8* and *EjMYB9* expression. Dual-luciferase assays indicated that EjMYB8, but not EjMYB9, could trans-activate promoters of lignin-related genes *EjPAL1*, *Ej4CL1* and *Ej4CL5*. Yeast one-hybrid assay indicated that EjMYB8 physically bind to *Ej4CL1* promoter. Furthermore, the putative functions of *EjMYB8* were verified using transient over-expression in both *N*. *tabacum* and loquat leaves, which increased lignin content. Moreover, combination of EjMYB8 and previously isolated EjMYB1 generated strong trans-activation effects on the *Ej4CL1* promoter, indicating that *EjMYB8* is a novel regulator of loquat fruit lignification.

## Introduction

Lignin is one of the main components required for plant secondary cell wall formation and its biosynthesis and transcriptional regulatory networks have been studied in various plants, particularly in *Arabidopsis* [1,2]. The synthesis of lignin monomers involve the phenylpropanoid pathway, initiated by L-phenylalanine ammonia-lyase (PAL), followed by Cinnamate 4-hydroxylase (C4H), 4-coumarate:coenzyme A ligase (4CL), Hydroxycinnamoyl CoA shikimate hydroxycinnamoyl transferase (HCT), Caffeoyl CoA 3-O-methyltransferase (CCoAOMT), Cinnamoyl CoA reductase (CCR), Caffeic acid 3-O-methyltransferase (COMT) and Cinnamyl alcohol dehydrogenase (CAD) [3].

Genes encoding enzymes of the phenylpropanoid pathway have been widely reported to be involved in controlling lignin biosynthesis, regulated by transcription factors, including MYB and NAC. For instance, at least 11 *AtMYB* genes were identified as regulators for lignin biosynthesis, including *AtMYB4*, *AtMYB26*, *AtMYB32*, *AtMYB41*, *AtMYB46*, *AtMYB58*, *AtMYB61*, *AtMYB63*, *AtMYB83*, *AtMYB85* and *AtMYB103* [2,4,5]. However, among these *AtMYB* genes, only *AtMYB58*, *AtMYB63* and *AtMYB85*, were identified as lignin-specific [6,7], while others were considered mainly to regulate secondary cell wall production. Similar transcriptional regulatory mechanisms have been uncovered in various woody trees, such as *Eucalyptus* and *Populus trichocarpa*, and *EgMYB2*, *PtrMYB3* and *PtrMYB20* are transcriptional activators [8], while *EgMYB1* represses lignin biosynthesis [9]. More lignin-related MYB transcription factors have recently been characterized from other plants, such as *PpMYB8* from pine tree [10] and *PdMYB221* from poplar [11].

MYB transcription factors could also interact with other transcription factors, such as members of the NAC transcription factor group. For instance, Arabidopsis *AtMYB83* is directly regulated by *SND1*, a NAC transcription factor [12], while *AtMYB26* up-regulated lignin related *NAC* genes, *NST1* and *NST2* [13]. There is some evidence that different MYB transcription factors participate in a transcriptional regulatory cascade, by regulating promoters of other MYBs, for instance *AtMYB46* binds to the secondary wall MYB-responsive element in the *AtMYB63* promoter [14]. Furthermore, MYBs could also form protein-protein complexes with other transcription factors and in *Arabidopsis*, MYB75 and KNAT7 interact to regulate secondary cell wall development in stems and seed coats [15].

Lignin accumulation occurs not only in model plants, woody trees and field crops, but is also important in some fleshy fruit, such as loquat (*Eriobotrya japonica* Lindl.) [16] and mangosteen (*Garcinia mangostana* L.) [17]. In different fruit, lignification occurred in different layers, in the flesh in loquat fruit and the pericarp in mangosteen. In loquat fruit, two MYB transcription factors were reported as regulators of loquat flesh lignification, with an activator type *EjMYB1* and repressor type *EjMYB2* [18]. Both *EjMYB1* and *EjMYB2* directly interacted with the promoter of the lignin-related biosynthetic gene *Ej4CL1*, and *EjMYB1* could trigger lignin accumulation in *N*. *tabacum* leaves [18]. In addition to MYB transcription factors, *EjNAC1* was characterized from loquat fruit, and shown to transcriptionally activate lignin biosynthetic genes. As with *EjMYB1*, *EjNAC1* over-expression enhanced lignin biosynthesis, however, the regulatory mechanisms involving *EjNAC1* remain unclear [19]. Most recently, an *AP2/ERF* gene, *EjAP2-1*, was also reported as an indirect regulator for loquat fruit lignification, via protein-protein interaction with EjMYB1 and EjMYB2 [20]. These transcription factors expanded the mechanistic understanding of the regulation of fruit lignification, but understanding is still limited compared to model or woody plants.

In the present study, twelve *EjMYB* genes were isolated from loquat fruit using RNA-Seq. Using the materials described in Xu *et al*. (2014), correlations between *EjMYB* and loquat fruit lignification were investigated and the transcriptional regulatory roles of *EjMYB* in controlling lignin biosynthetic genes were studied by dual-luciferase assay and yeast one-hybrid assay. Functional characterization of the key candidate, *EjMYB8*, was performed by transient overexpression in leaves of both *N*. *tabacum* and loquat seedlings. Furthermore, a significant additive transactivation of the *Ej4CL1* promoter was observed after *EjMYB1* and *EjMYB8* in combination.

## Materials and Methods

### Plant materials and treatments

Loquat fruit (*Eriobotrya japonica* Lindl. cv. Luoyangqing, ‘LYQ’) were harvested and bought from an orchard (name Butoutang) at Luqiao, Zhejiang province, China, at 2011. We confirm that the field studies did not involve endangered or protected species. The fruit were transported to the lab at Zhejiang University (Hangzhou, Zhejiang) at same day, for postharvest treatments. Three different treatments were set: the fruit were treated at 40°C (Hot air, 90–95% RH) for 4 h then transferred to 0°C storage (termed as HT); or pre-stored at 5°C for 6 d then transferred to 0°C storage (termed as LTC); and stored directly at 0°C (control). Details of treatments, sampling and physiological data (eg. Lignin content, firmness) were as previously described [18].

### Gene/promoter isolation and analysis

Differentially expressed *MYB*-related unigenes were selected, based on the annotations RNA-seq of ‘LYQ’ loquat fruit. The UTR and full ORF regions were amplified using a SMART^TM^ RACE cDNA amplification Kit (Clontech) and the primers are listed in S1 and S2 Tables. A phylogenetic tree for R2R3 type *MYB* was constructed by Fig Tree (version 1.4.2).

Regulatory roles of newly isolated *EjMYB* genes were investigated with both lignin biosynthetic genes and previous isolated transcription factors related to lignification. Promoters of loquat lignin biosynthesis related structural genes *EjMYB1* and *EjMYB2* were isolated in our previous reports [18,20]. Putative promoters of *EjAP2-1*, were isolated with a GenomeWalker kit (Clontech), using the primers described in S3 Table. The sequence of the putative promoter region is given in S4 Table.

### Real-time PCR analysis

For real-time PCR, gene specific oligonucleotide primers were designed and are described in S5 Table. The quality and specificity of each pair of primers was checked with melting curves and sequencing analysis [21]. *EjACT* (Genbank no. JN004223) was chosen as house keeping gene. Gene expression levels were expressed as a ratio relative to the fruit harvest time point (0 d, for fruit experiments) or empty vector (SK, for transient overexpression experiments), which was set to 1.

Total RNA extraction from loquat flesh and leaves used the published protocol [19]. cDNAs used for realtime PCR were synthesized using iScript^TM^ cDNA Synthesis Kit (Biorad).

PCR reactions were performed on a LightCycler 480 instrument (Roche). PCR reaction mixtures comprised 10 μl of LightCycler 480 SYBR Green I Master mix (Roche), 1 μl of each primer (10 μM), 2 μl diluted cDNA and 6 μl PCR grade water [22]. The PCR program was initiated with a preliminary step of 5 min at 95°C, followed by 50 cycles at 95°C for 10 s, 60°C for 10 s and 72°C for 15 s. Melting curve analysis were performed for each gene, at the end of each run.

### Dual-luciferase assay and yeast one-hybrid

Transcription factors recognize and regulate target promoters, which can be measured by dual-luciferase assays and yeast one-hybrid (Y1H) interactions [18,23]. Full-length transcription factors were cloned into pGreen II 0029 62-SK vector (SK), while the target promoter was inserted into pGreen II 0800-LUC vector [24]. Thus, full-length *EjMYB8* and *EjMYB9*, and the promoter of *EjAP2-1* were newly constructed, using the primers described in S3 and S6 Tables; while the other constructs were previously reported [18,20]. The dual-luciferase assays were performed in *N*. *benthamiana* leaves, according to protocols described in Zeng *et al*. (2015). Dual-luciferase assays were performed with at least three independent experiments (five biological replicates in each experiment).

According to the results of dual-luciferase assay, Y1H was further performed to verify physical binding of transcription factor and target promoter. Y1H was performed, using the Matchmaker^TM^ Gold Yeast One-Hybrid Library Screening System (Clontech, USA). Promoters of *Ej4CL1* was constructed into pAbAi vector by Xu *et al*. (2014), while the full-length *EjMYB8* sequence was subcloned into pGADT7 AD vector (primers are listed in S6 Table). TF-promoter interactions were tested on SD/-Leu containing 0-200ng/ml aureobasidin A (-Leu+AbA^200^) at 30°C for 3 d.

### Transient over-expression analysis of *EjMYB* in *N*. *tabacum* and loquat leaves

As loquat is a perennial woody fruit tree, it is difficult to perform stable transformation. In order to determine the roles of *EjMYB* genes, an unstable transient over-expression system, was adapted. Firstly, the transient expression analyses were performed in *N*. *tabacum*, using the same batch of *Agrobacterium* stock and infiltration protocol of the dual-luciferase assay. Target genes (MYB) and empty vector controls (SK) were infiltrated into two sides of the same leaves. Five days after infiltration, the infiltrated leaves (permeation ranges were recorded at infiltration) were sampled and used for lignin analysis. The transient expression analyses were repeated in at least three independent experiments (three biological replicates for each experiment). Similar experiments were also performed on young leaves of loquat seedlings. Transient over-expression experiments were repeated twice (three biological replicates in each). Five days after infiltration, the infiltrated leaves were sampled and used for lignin analysis

Transient over-expression experiments in both *N*. *tabacum* and loquat were conducted in a growth chamber (light: dark = 16 h: 8 h). Lignin content measurements were conducted according to the protocol described by Xu *et al*. (2014).

### Statistical analysis

The statistical significance of differences was calculated using Student’s *t*-test. Least significant differences (LSD_0.05_) were calculated using DPS7.05 (Zhejiang University, Hangzhou, China).

## Results

### Gene isolation and analysis

**Fig 1:**
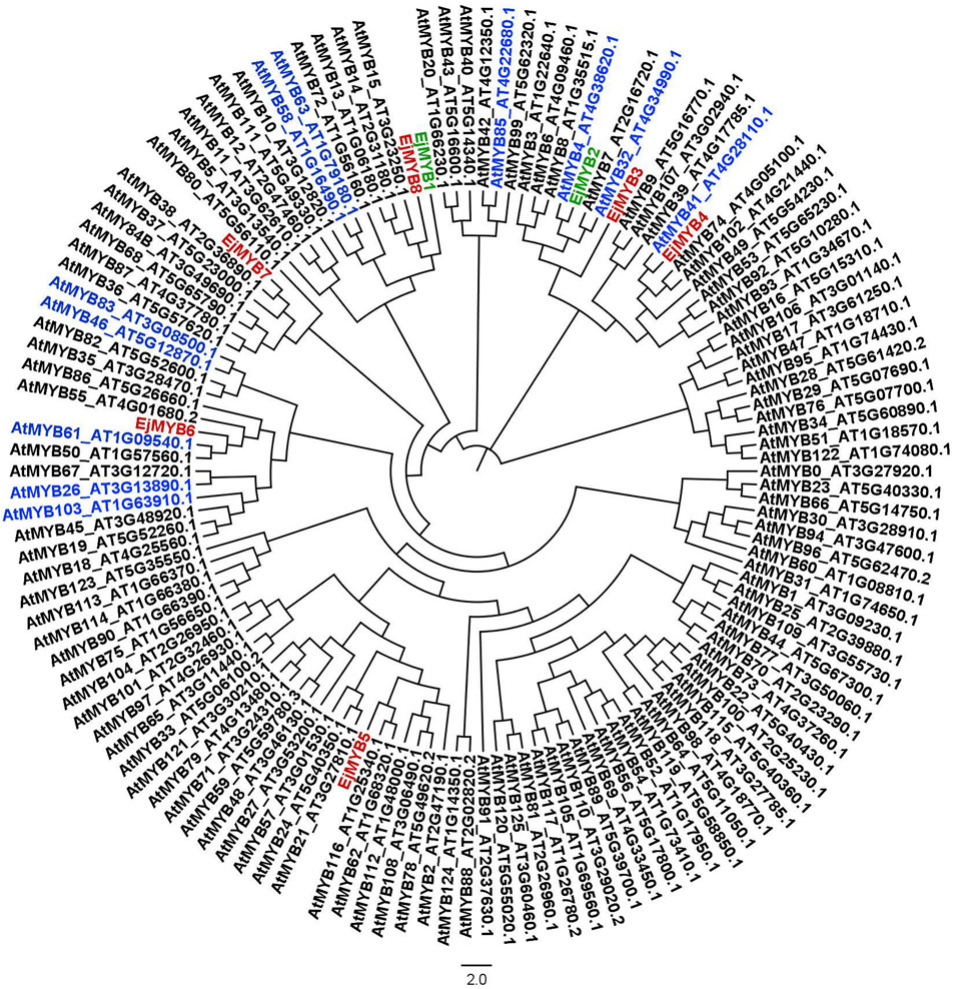
Phylogenetic analysis of R2R3 MYB transcription factors from loquat and Arabidopsis. Lignin-related AtMYBs are highlighted in blue and previously reported loquat EjMYB in green. Deduced amino acid sequences of *Arabidopsis* AtMYB were obtained from The Arabidopsis Information Resource (TAIR). Alignment was performed using the neighbor-joining (NJ) method in ClustalX (v. 1.81) and a phylogenetic tree was constructed with Fig Tree (v. 1.4.2).

Twelve *EjMYB* genes were isolated and designated as *EjMYB3-14* (Genbank no. KU534356-KU534367), in addition to the previously characterized *EjMYB1* and *EjMYB2* [18]. These *EjMYB* genes were of several different types with different MYB domain (S1 Fig), *EjMYB1-8* were R2R3 MYB, as indicated by possession of the conserved R2R3 domain (S2 Fig). Phylogenetic analysis that *EjMYB3* and *EjMYB8* were clustered with previously characterized *EjMYB2* and *EjMYB1* (Fig 1), while other *EjMYB* genes were in different sub-group, with *EjMYB6* in the same sub-group as the lignin-related *AtMYB46* and *AtMYB83* (Fig 1).

### Association between *EjMYB* expression and loquat fruit lignification

**Fig 2:**
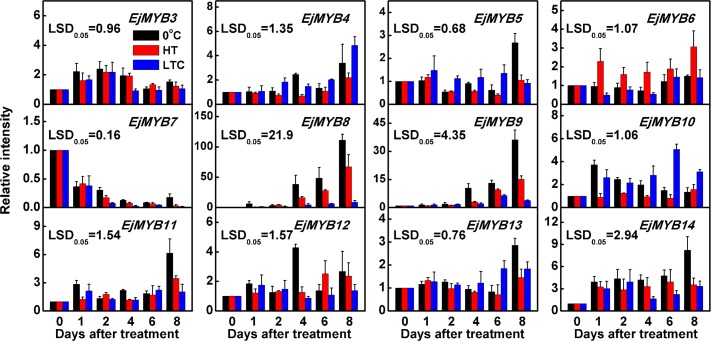
Expression of *EjMYB* genes in response to 0°C (Control), HT and LTC treatments. HT, heat treatment; LTC, low temperature conditioning. All of the materials were obtained from Xu *et al*., (2014). mRNA levels were expressed as a ratio relative to the harvest time point (0 d), which was set at 1. Error bars indicate S.E.s from three replicates.

Using the loquat fruit materials described in Xu *et al*. (2014), the expression patterns of 12 *EjMYB* genes were analyzed. Most of *EjMYB* genes showed increasing expression during low temperature (0°C) treatment, either during the whole storage period (eg. *EjMYB8*, *EjMYB9* and *EjMYB14*) or at specific sampling points (eg. *EjMYB5*, *EjMYB11* and *EjMYB13* at 8 d; *EjMYB12* at 4 d), However, only *EjMYB8* and *EjMYB9* were substantially repressed by HT and LTC treatments, which also alleviated, in which loquat fruit lignification, while the other low temperature-inducible *EjMYB* genes were much less responsive to HT and LTC treatments (Fig 2). *EjMYB8* and *EjMYB9* were also highly responsive to low temperature, and their mRNAs increased in abundance 111- and 36-fold, respectively. In contrast, transcript abundance of *EjMYB7* decreased during low temperature storage, and expression in HT and LTC treated fruit showed little change compared with low temperature stored fruit (Fig 2).

### Regulatory roles of EjMYB8 and EjMYB9 on lignin biosynthesis genes and related transcription factors

**Fig 3:**
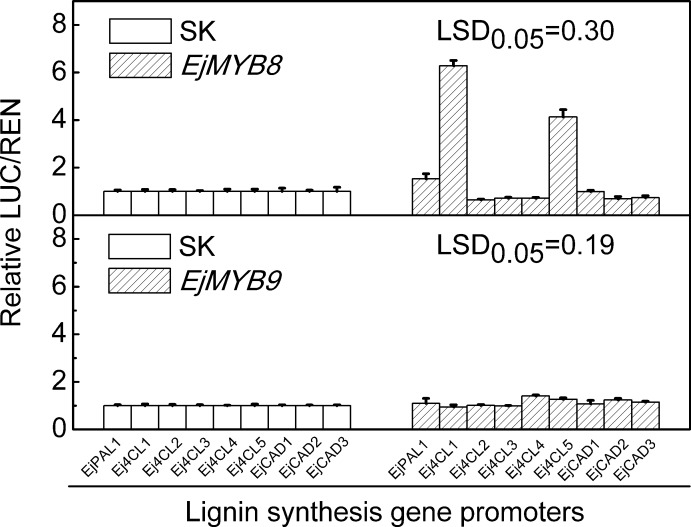
Regulatory effects of EjMYB8 and EjMYB9 on the promoters of lignin biosynthesis genes, using the dual luciferase assay. The ratio of LUC/REN of the empty vector (SK) plus promoter was used as calibrator (set as 1).

**Fig 4:**
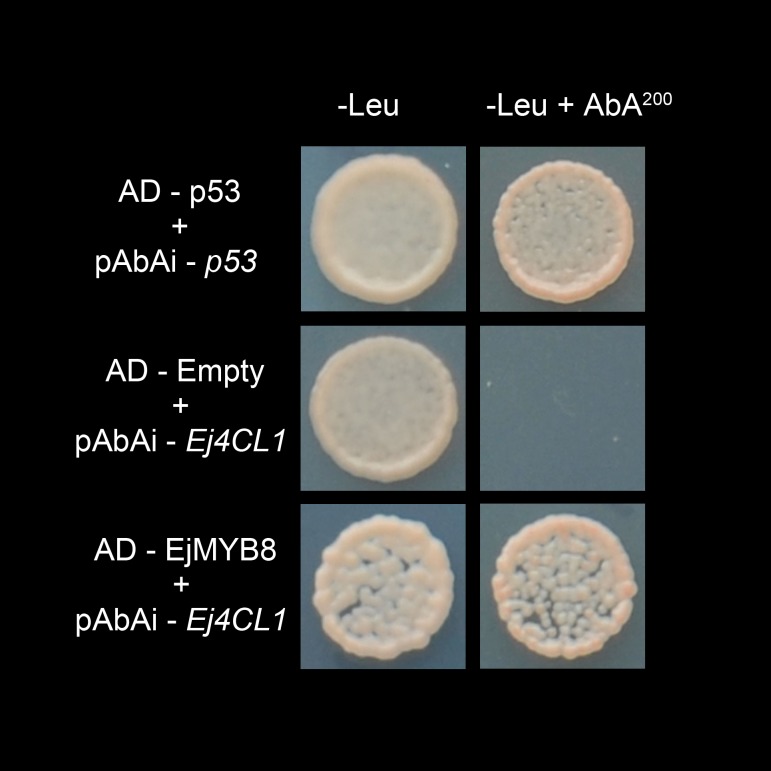
Yeast one-hybrid analysis of EjMYB8 binding to *Ej4CL1* promoter. Interaction was determined on SD medium lacking Leu in the presence of aureobasidin A (-Leu+AbA^200^). AD-p53 and pAbAi-*p53* were used as positive control; AD-empty and pAbAi-*Ej4CL1* were used as negative control.

EjMYB8 and EjMYB9 had particularly interesting expression patterns and were selected for further analysis. Dual-luciferase assays indicated that EjMYB8 significantly activated activities of *EjPAL1*, *Ej4CL1* and *Ej4CL5* promoters, with the *Ej4CL1* promoter being up-regulated about 6-fold, while *EjMYB9* had smaller effects (Fig 3). Y1H analysis indicated EjMYB8 could bind directly to the *Ej4CL1* promoter (Fig 4). These results indicated EjMYB8 could interact with and activate lignin biosynthetic genes. Possible regulatory roles of EjMYB8 on lignin related transcription factors were also investigated. However, dual-luciferase assay indicated that EjMYB8 was not able to influence promoters activities of the transcription factor genes *EjMYB1*, *EjMYB2* and *EjAP2-1* (S3 Fig), suggesting that EjMYB8 does not regulate these known lignin-related transcription factors in loquat.

### Functional characterization of *EjMYB8* in controlling lignin biosynthesis in both *N*. *tabacum* and loquat

**Fig 5:**
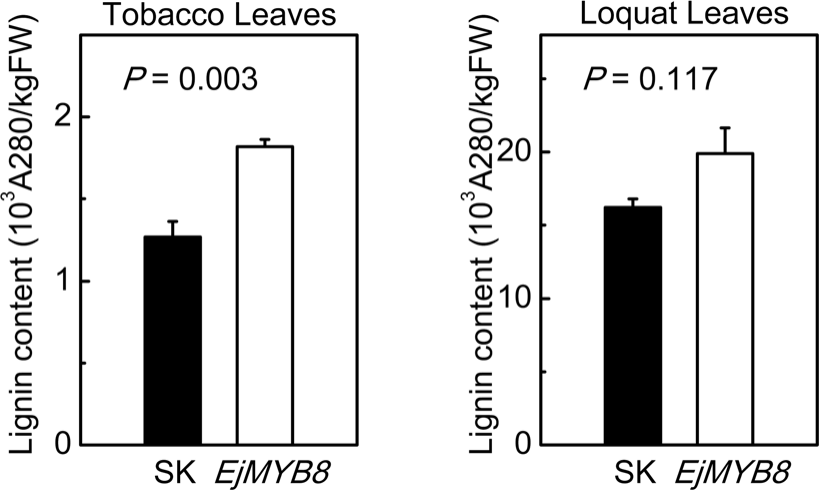
Transient over-expression of *EjMYB8* in *N*. *tabacum* and loquat leaves. The transient over-expression experiments were conducted with empty vector and *EjMYB8* on opposite sides of the same leaf. Error bars indicate S.E.s from three biological replicates. Student’s t-test was applied for the lignin content analysis and the significance levels indicated.

Due to the lack of a stable transformation system for loquat, transient over-expression approaches were adopted. *EjMYB8* transiently over-expressed in *N*. *tabacum* leaves significantly (*P*<0.01) promoted lignin accumulation, reaching 1.81×10^3^ A_280_kg^-1^ FW^-1^ compared with 1.26×10^3^ A_280_kg^-1^ FW^-1^ for the empty vector (Fig 5). Similar results were found in loquat leaves, where the half blades over-expressing *EjMYB8* had a higher lignin content of 19.8×10^3^ A_280_kg^-1^ FW^-1^, compared with the empty vector control half blades of 16.2×10^3^ A_280_kg^-1^ FW^-1^ (Fig 5).

### Combination effects of EjMYB8 and EjMYB1

**Fig 6:**
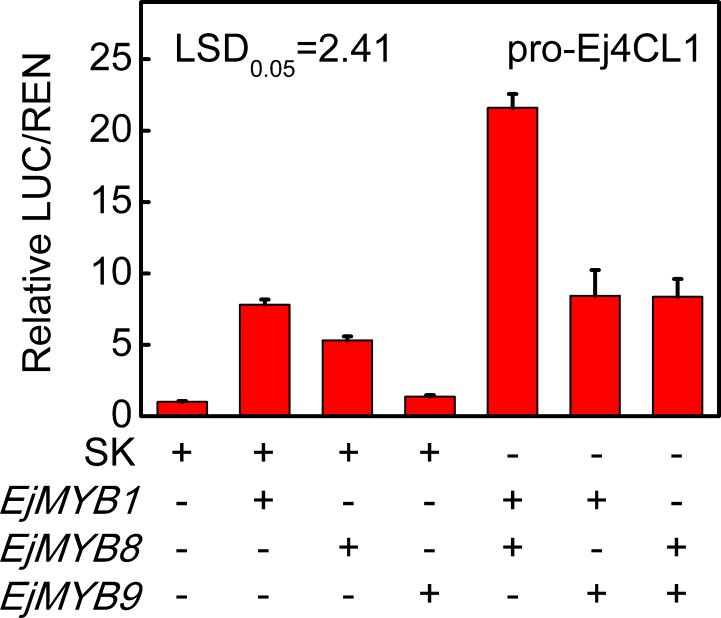
Synergistic trans-activation of the *Ej4CL1* promoter by combination of EjMYBs. The ratio of LUC/REN of the empty vector (SK) plus promoter was used as calibrator (set as 1). + and—means presence and absence of indicated constructs. Error bars indicate S.E.s from five replicates. LSDs represent least significant difference at *p* = 0.05.

The results of gene expression, dual-luciferase assay, Y1H and transient over-expression indicate that EjMYB8 is an activator of loquat fruit lignification. It promotes transcription from the promoters of lignin biosynthesis genes, as found for the previously reported transcriptional activator, EjMYB1 using dual-luciferase assay [18]. EjMYB1 and EjMYB8 together caused a substantially higher induction (approximate 21 folds) of expression from the *Ej4CL1* promoter (Fig 6). The results of yeast two hybrid assays, however, indicated that EjMYB8 could not directly interact with EjMYB1 (S4 Fig).

## Discussion

Fleshy fruit lignification is important both commercially and scientifically. For industry, lignification adversely influences fruit storability and quality, for instance, loquat fruit lignification is usually accompanied by flesh browning reduced juice extractability. Thus, many artificial technologies have been developed to alleviate lignification, such as LTC [16], 1-MCP [25], HT [18], Methyl Jasmonate (MeJA) [26]. Mechanisms controlling fruit lignification are particularly interesting, as it occurs in productive edible organs, not vegetative organs of model plants. The underlying regulatory mechanisms of low temperature induced lignification and the technologies developed to alleviate it remain generally unknown.

Previous research has indicated that *EjMYB1* and *EjMYB2* transcriptionally regulate loquat fruit lignification, and both *EjMYB* genes were cloned based on their similarity to *Arabidopsis* lignin regulators *AtMYB58* and *AtMYB4* [18]. We investigated whether there could be additional *EjMYB* genes involved in loquat lignification. Twelve *EjMYB* were isolated based on RNA-seq and RACE. Phylogenetic analysis suggested, that other R2R3 type *EjMYB* genes could be involved in loquat fruit lignification, as they were clustered with known lignin-related MYB, such as *AtMYB4*, *AtMYB46* and *AtMYB58* [7,27,28]. However, gene expression indicated that only *EjMYB8* and *EjMYB9* (not R2R3 types) were significantly correlated with loquat fruit lignification, as their transcripts abundance were significantly prohibited by LTC and HT treatment, which reduced lignification.

A role for *EjMYB8* was confirmed by the demonstration that it had the ability to trans-activate promoters of lignin biosynthesis related genes (*EjPAL1*, *Ej4CL1* and *Ej4CL5*) from loquat fruit and Y1H assay indicated a physical interaction between *EjMYB8* and the *Ej4CL1* promoter. On contrast, *EjMYB9* could not regulate these promoters. Significantly, all results for *EjMYB8*, including gene expression, dual-luciferase assay and Y1H, mimicked those for *EjMYB1* [18] and *EjMYB8* was clustered with *EjMYB1*. At least two *AtMYB* genes, *AtMYB58* an *AtMYB63*, which fall in the same sub-group as *EjMYB1* and *EjMYB8*, have also been characterized as regulators of lignin biosynthesis [7], suggesting multiple MYB family members may have similar function. However, *EjMYB8* encoded a novel protein, with only 52% amino acid sequence identity to *EjMYB1* (data not shown), indicating that, *EjMYB8* is a novel *MYB* transcription factor that participates in loquat fruit lignification, via transcriptional regulated of structural genes. The function and regulatory mechanisms of *EjMYB9* on loquat fruit lignification remain unknown, which might not a direct regulator on lignin biosynthesis genes and, require further investigation.

The function of *EjMYB8* was verified in *N*. *tabacum* and loquat leaves. For perennial fruit, functional verifications are generally a bottleneck, due to the lack of stable transformation systems and transient expression systems have been widely adopted for functional analysis of fruit genes, such as *MdMYB10* for apple anthocyanin regulation [29]; *PpMYB10*.*4* for peach anthocyanin biosynthesis [30]; *AdGT4* for kiwifruit aroma [31]. Here, transient over-expression of *EjMYB8* in loquat and *N*. *tabacum* supported the role of *EjMYB8* as a regulator of loquat fruit lignification.

MYB transcription factors have been widely reported to be involved in many aspects of plant metabolisms, via interaction with other transcription factors, such as MYB-bHLH-WD40 for anthocyanin regulation [32,33] and also MYB-ZML for lignin biosynthesis [34]. In loquat, protein-protein interactions have also been observed between lignification related transcription factors EjMYB1/2 and EjAP2-1 [20]. However, both dual-luciferase and yeast two hybrid assays indicated that EjMYB8 could not interact with the EjMYB1 protein or promoter (S3 and S4 Figs). Thus, the underlying mechanisms of the addictive effects of *EjMYB1* and *EjMYB8* require further investigation.

## Conclusions

Twelve *EjMYB* genes were isolated from loquat fruit and *EjMYB8* was characterized as a novel activator for loquat fruit lignification, according to the results of gene expression and dual-luciferase assay. Y1H indicated EjMYB8 could directly interact with *Ej4CL1* promoter. Furthermore, *EjMYB8* and *EjMYB1* acted synergistically to enhance expression of *Ej4CL1*. The present study has thus identified a novel MYB transcription factor (*EjMYB8*) and possible MYB-MYB linkage for loquat fruit lignification.

## Supporting Information

S1 Fig Distribution of conserved domains in EjMYB protein sequences. (TIF)

S2 Fig Alignment of R2R3 domains from EjMYB transcription factors. (TIF)

S3 Fig Regulatory effects of EjMYB8/9 on the promoters of lignification-related transcription factors, using the dual luciferase assay. (TIF)

S4 Fig Yeast two-hybrid assay for EjMYB1 and EjMYB8. (TIF)

S1 Table Primes sequences for 5’-RACE analysis. (DOCX)

S2 Table Primer sequences for 3’-RACE analysis. (DOCX)

S3 Table Primers for *EjAP2-1* promoter isolation and vector construction. (DOCX)

S4 Table Sequence of *EjAP2-1* promoter. (DOCX)

S5 Table Primers for Real-time PCR. (DOCX)

S6 Table Primers for EjMYB full-length sequences clone. (DOCX)

## Abbreviations

AbA: Aureobasidin A
cv: Cultivar
FW: Fresh weight
HT: Heat treatment
LSD: Least-significant difference
LTC: Low temperature condition
LUC: Firefly luciferase
ORF: Open reading frame
QPCR: Real-time quantitative PCR
RACE: Rapid amplification of cDNA ends
REN: Renilla luciferase
RH: Relative humidity
SD: Synthetic dropout
TFs: Transcription factors
UTR: Untranslated region
Y1H: Yeast one-hybrid
1-MCP: 1-methylcyclopropene
