# Supplementary figures and images for: EjMYB8 Transcriptionally Regulates Flesh Lignification in Loquat Fruit
Source: PLoS One. 2016 Apr 25;11(4):e0154399. doi: 10.1371/journal.pone.0154399 (PMC4844104; doi:10.1371/journal.pone.0154399)

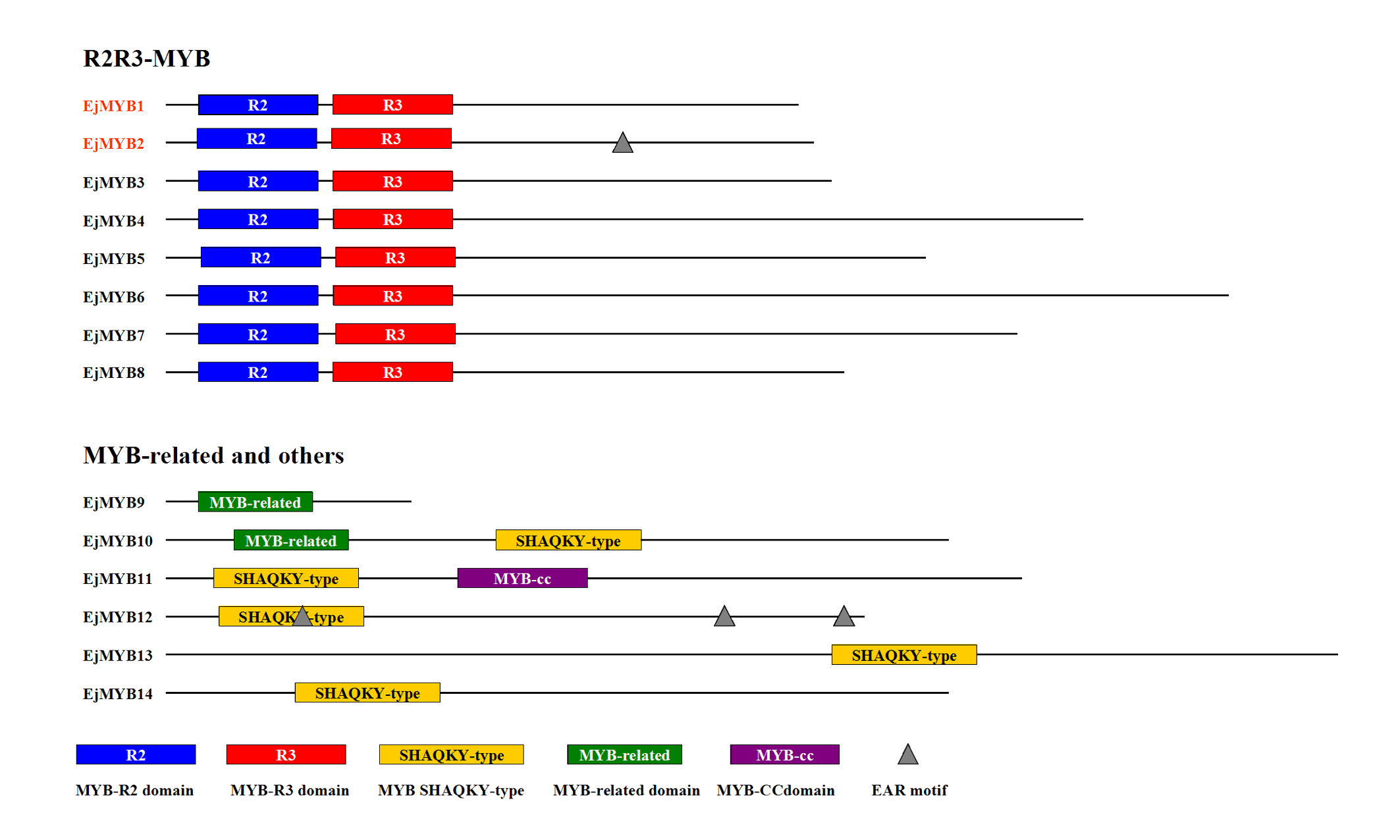

Supplement: S1 Fig — (TIF) [file pone.0154399.s001.tif]

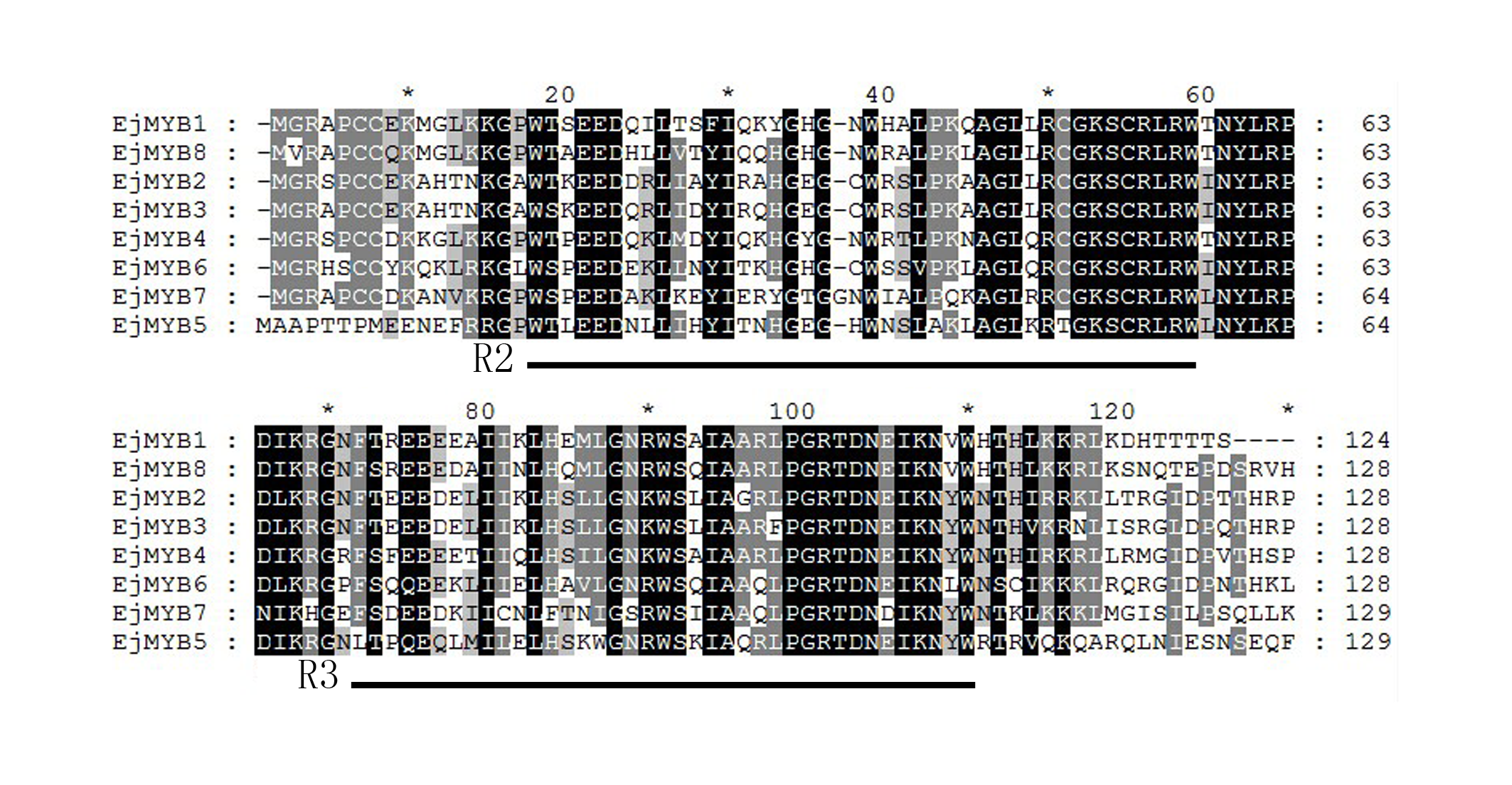

Supplement: S2 Fig — (TIF) [file pone.0154399.s002.tif]

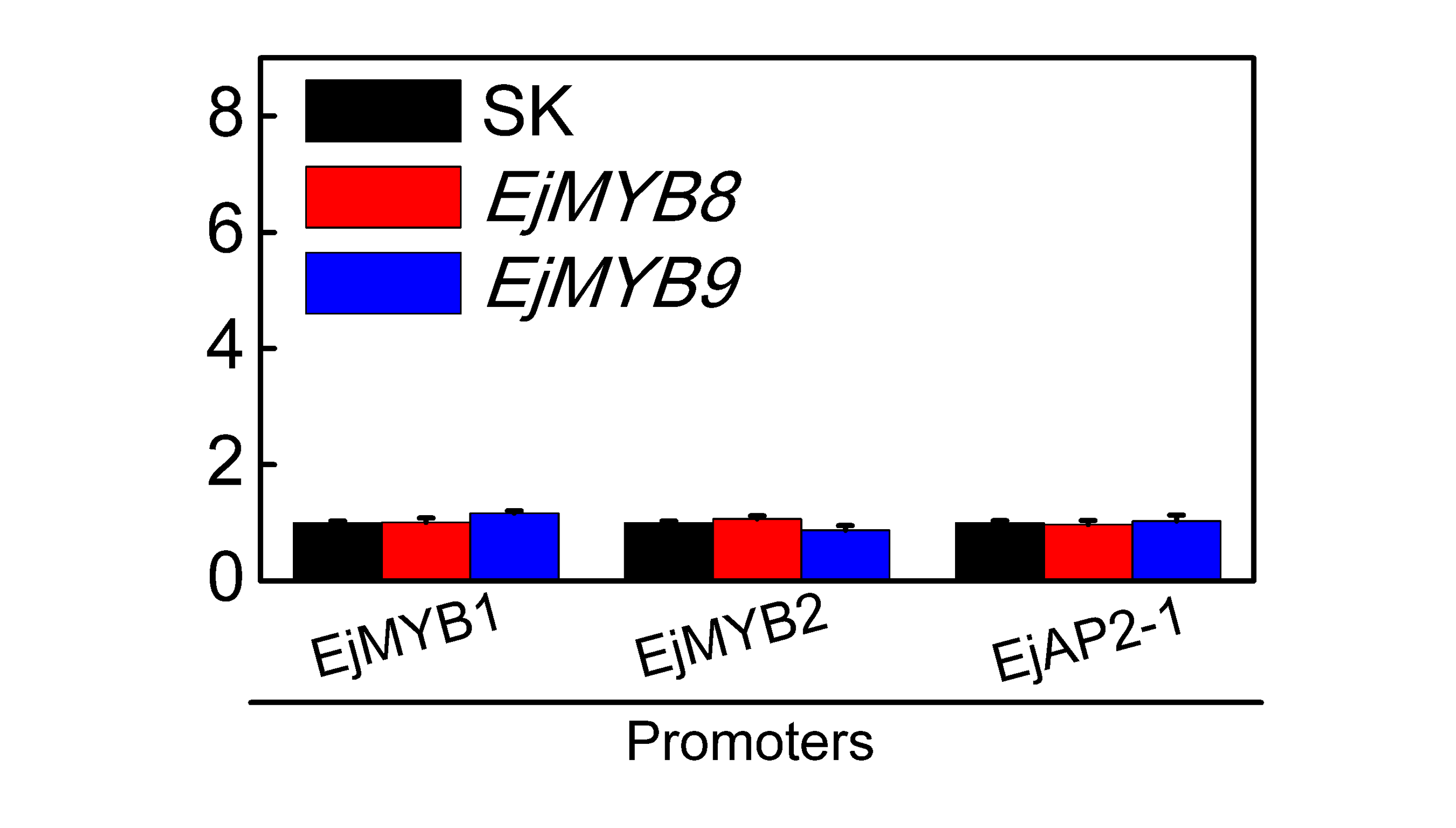

Supplement: S3 Fig — (TIF) [file pone.0154399.s003.tif]

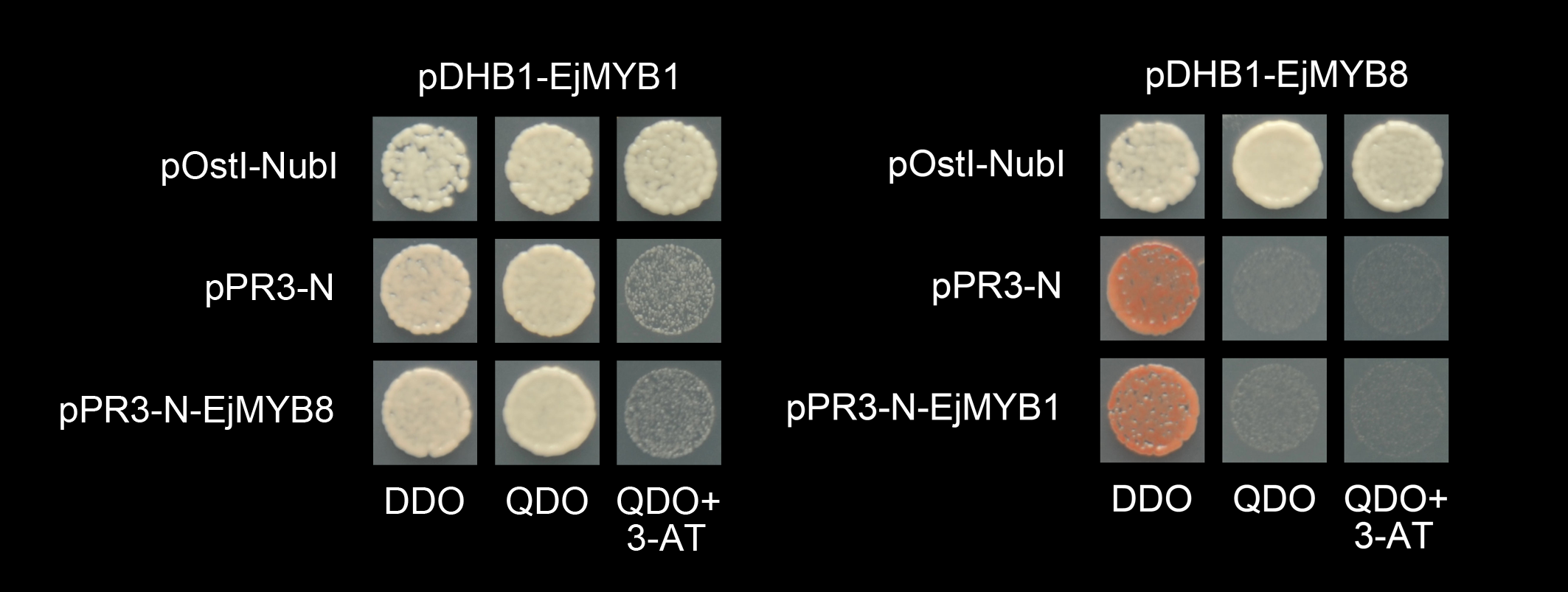

Supplement: S4 Fig — (TIF) [file pone.0154399.s004.tif]
