# Supplementary material for: EjMYB8 Transcriptionally Regulates Flesh Lignification in Loquat Fruit
Source: PLoS One. 2016 Apr 25;11(4):e0154399. doi: 10.1371/journal.pone.0154399 (PMC4844104; doi:10.1371/journal.pone.0154399)
Supplement: S1 Table — (DOCX) [file pone.0154399.s005.docx]

**Supplemental Table 1** Primes sequences for 5’-RACE analysis

| *Gene* | *GSP1 (5′ to 3′)* | *NGSP1 (5′ to 3′)* |
| --- | --- | --- |
| *EjMYB3* | GCTCTGAAATTCAACCTGGCAGCAC | CACCGCCGACGACCGCACTTTTT |
| *EjMYB4* | GCTCTGGGTCGAGTCCAATTGGTTG | ACGGCAGCTCTTCCCACATCTTTGT |
| *EjMYB5* | TCGGTAAAATGCAGGGGGTGAACTC | ATGCGGAGTTCTGAGAAGGGCTCAA |
| *EjMYB6* | TTATCGGATGGGAGGCTGATGGAAG | TGTGAGTGTTGGGGTCAATGCCTCT |
| *EjMYB7* | AGAAATGGGCTCCGAAGTGTTGGTG | TTTAGCAGCTGGGATGGGAGAATGC |
| *EjMYB8* | GTTGTTGTTGTCGTCGTCGCTCGTT | TGGGCTCTAGCTCTTGCTCCGTTTT |
| *EjMYB9* | TGTCTACCGCTGGAGGGTGTCTTCC |  |
| *EjMYB10* | GCGATCCATGAAGATTGCCACTCAG | CTGCTGCACCACAGTTGAGGAGTGA |
| *EjMYB11* | ACGCCTTATTGTCCGGGTGAATGTC | TGCTTCCGCTCCATGTCTTTTAGGG |
| *EjMYB12* | CATCCTCGGCCTCTTCAAACGAATG | CTTTTTATGGCCTCCGAGCCTTTCG |
| *EjMYB13* | CGTTTCTCTGATCTCCGCCGTTCAT | GACTTGCCGTCTTCGATGCTCTCTG |
| *EjMYB14* | CGTTGCCTGTCCCTCATGTCTTCAG | GCCGAACGAGTTTCTTCGCGTAATG |
