# Supplementary material for: EjMYB8 Transcriptionally Regulates Flesh Lignification in Loquat Fruit
Source: PLoS One. 2016 Apr 25;11(4):e0154399. doi: 10.1371/journal.pone.0154399 (PMC4844104; doi:10.1371/journal.pone.0154399)
Supplement: S2 Table — (DOCX) [file pone.0154399.s006.docx]

**Supplemental table 2** Primer sequences for 3’-RACE analysis

| *Gene* | *GSP2 (5′ to 3′)* | *NGSP2 (5′ to 3′)* |
| --- | --- | --- |
| *EjMYB3* | AGCCGTTTCAGTCCAAACCAACTCG | CAGGTTGAATTTCAGAGCAGCGAAGC |
| *EjMYB4* | CCAACCAATTGGACTCGACCCAGAG | ATCATGCAGCAGCAGCCCAATGTAG |
| *EjMYB5* | GGACCGGAAAAAGCTGCAGATTGAG | CTCCGCATCTCCTTCTCTGTCACCA |
| *EjMYB6* | GAGAAGGCCTCTGTGGGATCCAATG | AGAGTTCCTGCAACCGTCCATGTCA |
| *EjMYB7* | GCATTCTCCCATCCCAGCTGCTAAA | CACCAACACTTCGGAGCCCATTTCT |
| *EjMYB8* | AGCAGGGAAGAAGAAGACGCCATCA | CATATGTCACAACGAGCGACGACGA |
| *EjMYB9* | GGGAAACCCCTGATCGTTGGTGTAA | CTCCAGCGGTAGACACAGCACCAAT |
| *EjMYB10* | AAGGGGTGCCATGGACAGAAGAAGA | GAAGTTGGCCAGCATGAGCATTGAC |
| *EjMYB11* | GAACTTGCAAGGGCCGAACTCACTC |  |
| *EjMYB12* | CATTCGTTTGAAGAGGCCGAGGATG | AGGATGCTCACCCTTCAGCCTTCAA |
| *EjMYB13* | CCACACTCTCCAACACCAGCTCCAC | TCGGATAGCAGGAGCTGGAAGGGTA |
| *EjMYB14* | GCAGTGGAAAGTTCAGGAGAGAAGCA | CTGCAGTTCACGGAAGGGGTTTGTG |
