# Supplementary material for: EjMYB8 Transcriptionally Regulates Flesh Lignification in Loquat Fruit
Source: PLoS One. 2016 Apr 25;11(4):e0154399. doi: 10.1371/journal.pone.0154399 (PMC4844104; doi:10.1371/journal.pone.0154399)
Supplement: S3 Table — (DOCX) [file pone.0154399.s007.docx]

**Supplemental Table 3** Primers for *EjAP2-1* promoter isolation and vector construction

| *Gene* | *Method used* | *GSP1 (5′ to 3′)* | *GSP2 (5′ to 3′)* |
| --- | --- | --- | --- |
| *EjAP2-1* | *Genome walking* | ATCAGACGAAGAAGAAGATCAGGACGT | TTAATGGTCGGTTCCATGGTTCACATT |
