# Supplementary material for: EjMYB8 Transcriptionally Regulates Flesh Lignification in Loquat Fruit
Source: PLoS One. 2016 Apr 25;11(4):e0154399. doi: 10.1371/journal.pone.0154399 (PMC4844104; doi:10.1371/journal.pone.0154399)
Supplement: S4 Table — (DOCX) [file pone.0154399.s008.docx]

**Supplementary Table 4.** Sequence of *EjAP2-1* promoter

| *Gene* | *Promoter sequences (5′ to 3′)* |
| --- | --- |
| *EjAP2-1* | CGACGGCCCGGGCTGGTACTTGATTTTAATAGAAGACTTGTCGTACTTGGATCTATCAGAAGACTTGGCAAAAAACGAGCGTAGTAGCGGTTAAGAATTTATATAGCCGACCCTGCTTAGTAGAATAAAGTTTTGTTATTATATCATTCTAATTATATAATGATATGTGATATTATGTTCCGTGTTTTAAGCACACCGAAAATTCTCGCGGGTAGAGGAAGGGAGAGTGGCTTGTGACGTGAACGTTGAATAATTATGTAGCCAATAAAGGTAATTGAGTCTTTGGGTTGTCTATTTCCATTAGTGGGTAAGAATAGAGAGAGAAAGAATTGAGAGAATACTTTTCGATTTGGTTAACGTGGGAAAGTTGTGTAAAGAAAGAGACGGTTCTTCTTTTTTTTTTTTTTTTTTTTTCCTCCCATTTCCCCATCTGATCATGCTCCTTAATATGATCAAACCACCAATAAAATAAAAAATAAAAAAATAAAAAATAAAAAATACTGTGACATTAATATAACAATCTAATGTAATTTTAATCCGCTTACAAGTAAAAAGATTTAGATTCGATTTTTATGAATGATTAATTTGAGATCAGATTATCATGGTTAGAGAGAGAAACAGCGAATAGTAAAGTTGTACTAAACCCAACAGGTTCAACTAAAGAAAAATGAGAAGGAAAAAAAGAAAAAGAAAGAAACTTGGGTCTTTATTTAATATAGATGCCAAAAATACATACTACAATTTATTTTATTTTATTTTAATAAAAATGACTGATTTGCATAAAATAAAAAGAAACAAAAACAAGAAAGATTGAGAGAGAGTGAACTATATGTCATTGAAAAGAAAGGAAGGAAAGTTGTTAGAGAGAGAGAGGTATTAACATTAGGGGTGTGGTGTGGTGGGATGCTTGAGCTAAAGCTTTGCTGAAAACCGGCCTCGTCTATGTTGTATTGGGCAGTTTAGGCGCCATTCCAGCACAAGATCTTGCATCACCTCTACCTCTCTCTCTCTCTCTTTATAGCTCTCTAGCTATCTTTTTCTCTCTGGACTTTCATCAAACACAACTACATCACTAACCTCCTCTCTCTCTAACTTTTTTTCTTCATGTTTCTTTGCTCAAGTTGAAGCTTTTTGCCACGCTGAATTCTGGGTTTAATGTGAAAA |
