# Supplementary material for: EjMYB8 Transcriptionally Regulates Flesh Lignification in Loquat Fruit
Source: PLoS One. 2016 Apr 25;11(4):e0154399. doi: 10.1371/journal.pone.0154399 (PMC4844104; doi:10.1371/journal.pone.0154399)
Supplement: S5 Table — (DOCX) [file pone.0154399.s009.docx]

**Supplemental Table 5** Primers for Real-time PCR

| *Gene* | *Forward primer (5′ to 3′)* | *Reverse primer (5′ to 3′)* |
| --- | --- | --- |
| *EjACT* | CCAGGGCTGTGTTTCCTAGT | CATGTCATCCCAGTTGCTCA |
| *EjMYB3* | CAGCAGAAATACAAGTGTGGTGA | TGATCTGCTGTAGTCTTGACTCG |
| *EjMYB4* | CGCCAGAGACTTCAAATATTCAC | AAGATGCTTGGGATGTCAAACT |
| *EjMYB5* | GGCTACAACAGCTTAAGTCCAGA | CCAGGTTCCATAAATTGTCAGTC |
| *EjMYB6* | CCAGAAGAGATCAAATGGTCTGA | TGTGTGACTGAGATTCTGGTTTG |
| *EjMYB7* | GAACAAGACACAGGAAAAGGTCA | CCCTCTCTCTCTCTCTCCAAAAA |
| *EjMYB8* | AAGTGAATATGGGGCCTACTGAT | AGCAATTCTGGAATTTCCTCAG |
| *EjMYB9* | GACGACTGAAGAGGAAGTGAAGA | CCAACTTTCTTATACTTGGGAAGG |
| *EjMYB10* | TGGGATGTCTTCACATGTACCTA | TGGAATAGTACACCCTCTTCTGG |
| *EjMYB11* | CCGAAAGCAATAGACTTGAACTG | AGGACTGGAGGGAATTATGTTTG |
| *EjMYB12* | GAATCGGAGCCTTAATCTGAATC | AGAAACAGAATGTGGGGTGAGT |
| *EjMYB13* | ATAGCAGGAGCTGGAAGGGTACT | CATCAAAAGGTCAGAACTTGAGC |
| *EjMYB14* | CAGACGGAAAGATCATTTGGTAG | TGTCCCTCATGTCTTCAGAAACT |
