# Supplementary material for: EjMYB8 Transcriptionally Regulates Flesh Lignification in Loquat Fruit
Source: PLoS One. 2016 Apr 25;11(4):e0154399. doi: 10.1371/journal.pone.0154399 (PMC4844104; doi:10.1371/journal.pone.0154399)
Supplement: S6 Table — (DOCX) [file pone.0154399.s010.docx]

**Supplemental Table 6** Primers for *EjMYB* full-length sequences clone

| *Gene* | *Purpuose* | *Primers* | |
| --- | --- | --- | --- |
| *EjMYB8* | Construct to pGreen SK vector | FP | ATGGTTAGAGCTCCTTGCTGCCAGA |
|  |  | RP | TCAAATTCCTAGCAATTCTGGAATT |
|  | Construct to pGADT7 AD vector | FP | CAAGGCCATGGAGGCCATGGTTAGAGCTCCTTGCTGCCAGA (*Sfi I*) |
|  |  | RP | TCTGGATCCTCAAATTCCTAGCAATTCTGGAATT (*BamH I*) |
| *EjMYB9* | Construct to pGreen SK vector | FP | ATCGCGGCCGCATGGCACCTAGCAAGCTTTCTTCC (*Not*I) |
|  |  | RP | TGTACTAGTTTAGAGCTTTAGGGTCTTTAGCC (*Spe*I) |
